# Non-Pharmacological Management of Idiopathic Pulmonary Fibrosis

**DOI:** 10.3390/jcm14041317

**Published:** 2025-02-17

**Authors:** Jon B. Mullholand, Catherine E. Grossman, Apostolos Perelas

**Affiliations:** Division of Pulmonary Disease and Critical Care Medicine, Virginia Commonwealth University, Richmond, VA 23298, USA; catherine.grossman@vcuhealth.org

**Keywords:** idiopathic pulmonary fibrosis (IPF), interstitial lung disease (ILD), supplemental oxygen, lung transplant, pulmonary rehabilitation, sleep, diet, palliative care, GERD, lung cancer

## Abstract

Idiopathic pulmonary fibrosis (IPF) is a relatively common progressive fibrotic interstitial lung disease associated with significant morbidity and mortality. The available medications for IPF only slow down the disease process, with lung transplantation the only option for a cure. Non-pharmacological therapies are significant adjuncts that can improve symptom burden and quality of life with minimal or no side effects. Supplemental oxygen can improve exercise capacity and the sensation of dyspnea in a significant portion of patients with resting or exertional hypoxemia and has been supported by several professional societies. Pulmonary rehabilitation is a comprehensive program that includes education and therapeutic exercises to improve patient stamina and strength. It is one of the few interventions that have been shown to produce a meaningful increase in a patient’s exercise capacity, but its wide adoption is limited by availability, especially in rural areas. Sleep optimization with supplemental oxygen and positive airway pressure therapy should actively be investigated for all patients diagnosed with IPF. Although gastroesophageal reflux control with non-pharmacological means is still controversial as an intervention to reduce the rate of lung function decline, it can help control reflux symptoms and improve cough intensity. IPF patients should be educated on the importance of balanced nutrition and the potential benefits of screening for lung transplantation. Palliative medicine can help with symptom control and should be considered for all patients regardless severity, but especially in those in the later stages of disease.

## 1. Introduction

Idiopathic pulmonary fibrosis (IPF) is a relatively common progressive fibrotic interstitial lung disease (ILD) associated with significant morbidity and mortality [1]. Despite the progress made in understanding its pathogenesis, the therapies available to date have only been shown to reduce the rate of disease progression without a major effect on symptom burden or quality of life [2,3]. Moreover, they are associated with a significant side effect risk and poor tolerance [4]. Consequently, lung transplantation remains the only definitive therapy for IPF, but it is plagued by high costs, limited donor availability, and suboptimal long-term outcomes [5]. This review will focus on non-pharmacological therapies for IPF; many of these interventions are directed towards symptoms and have been shown to improve quality of life with minimal or no side effects.

## 2. Supplemental Oxygen

IPF results in hypoxia through a variety of mechanisms; diffusion impairment, ventilation/perfusion mismatch, and pulmonary vasculopathy have all been demonstrated to be present and contribute to the phenomenon in varying degrees [6]. Hypoxia can initially be seen only during sleep or heavy exertion, but it eventually occurs at rest, and it has been associated with reduced exercise capacity, poor quality of life, and reduced survival [7,8]. It is thus not surprising that oxygen therapy is one of the key criteria for consideration for lung transplantation according to the latest guidelines [9]. Most patients with IPF will require supplemental oxygen at some time during their disease, and for most of them, it represents a major milestone in their life.

Despite its frequent use, there are surprisingly few quality data to guide strategies around oxygen supplementation in IPF, with most clinicians and even society guidelines relying on COPD data [10]. However, time after time, it has been shown that patients with IPF are different than those with COPD; in general, they tend to have a higher prevalence of hypoxemia, an increased degree of desaturation, and increased needs for supplemental oxygen to avoid exertional hypoxemia. The 2020 ATS guidelines, although succinct, were a very good initial step in bringing more attention to the practice of supplemental oxygen prescription for this population while raising awareness of the need for liquid oxygen [10].

Long-term oxygen therapy describes supplemental oxygen for at least 15 h a day. It has been advocated for patients with severe resting hypoxemia based on the most recent ATS guidelines [10] despite the lack of evidence supporting a mortality or quality of life benefit [11]. It has been recommended for patients with PaO2 of ⊆ 55 mmHg or SpO2 of 88% or below at rest or in those with PaO2 55–59 mmHg or SpO2 89% with evidence of right ventricular dysfunction [10]. Ambulatory oxygen has been recommended in patients with severe hypoxemia while ambulating on room air [10]. The current evidence does not suggest a benefit in mortality, but supplemental oxygen has been shown to improve quality of life metrics, dyspnea, and exercise capacity among patients with IPF [12,13], although not in all patients [14].

High-flow oxygen has been examined in patients exercising, with promising results to date. Two trials have examined high-flow oxygen and compared it to the standard of care; Badenes-Bonet et al. demonstrated an increase in exercise tolerance during a CPET [15], whereas Harada et al. demonstrated increased ability to perform submaximal exercise with less desaturations and fatigue [16]. A meta-analysis showed that during exercise, high-flow oxygen systems had better outcomes than low-flow systems, and, among high-flow systems, nasal cannula outperformed venturi devices [17].

Traditionally, supplemental oxygen needs have been calculated with titration during a 6 min walk test. However, there are emerging data suggesting that this may underestimate the patient’s real needs [18]. There are currently many commercially available wearable portable oximeters; however, so far, they have not been used to assist with accurate supplemental oxygen prescription. Continuous oxygen flow is preferred over pulse flow, especially for those with higher oxygen needs, and most patients will get the most benefit by using oxygen tanks despite their greater weight and reduced portability [10]. Liquid oxygen has been proposed for patients requiring >3 lt via NC through continuous flow; however, availability remains very limited, which is mostly driven by costs and poor reimbursement by insurance companies [10].

## 3. Pulmonary Rehabilitation

Pulmonary rehabilitation (PR) is “a comprehensive intervention based on a thorough patient assessment followed by patient-tailored therapies that include, but are not limited to, exercise training, education, and behavior change, designed to improve the physical and psychological condition of people with CRD and to promote the long-term adherence to health-enhancing behaviors” [19]. PR has been a staple of treatment for patients with advanced lung disease, including, in particular, individuals with COPD [20].

A recent meta-analysis of nine studies revealed a moderate increase in exercise capacity [estimated based on the 6 min walk distance (6 MWD)] and an improvement in dyspnea scores [21]. The improvement in the 6 MWD (37.25 m) reached the minimal clinically significant improvement for patients with fibrotic lung disease [22]. Additionally, there was a noted benefit in HRQoL (Health-Related Quality of Life), which was determined using the IPF-specific SGRQ-I (St. George Respiratory Questionnaire—Idiopathic Pulmonary Fibrosis) [21]. Unfortunately, neither of these benefits were sustained in the IPF-specific sub-group at the 6–12-month follow-up in contrast to the general ILD group. This led to an update in the ATS/ERS guidelines for a strong recommendation for the use of PR in ILD; however, no specific recommendations were made for IPF [20]. Moreover, a recommendation was made for repeating PR in regular intervals to sustain the benefits [23]. In practice, and given the limitations in the availability of PR, this is achieved with the help of maintenance programs, which the patients can join after completing PR.

Even though there are data for general use of PR in IPF, there are still many questions left as to the best model to provide PR. First is the role of supplemental oxygen during PR. Whereas most programs titrate supplemental oxygen based on a 6 min walk test to maintain SpO2 of 89% or above, there is evidence that high-flow oxygen may result in increased duration of exercise tolerance, along with less fatigue and less desaturations [16]. In a recent review of high-flow oxygen in the setting of PR, it was noted that these benefits are inconsistent across the board of ILD, most likely due to high heterogeneity when investigating the benefits [24]. There is an active multi-center trial in Canada studying the role of high-flow oxygen for pulmonary rehabilitation in individuals with clinically stable IPF (NCT02551068) [25]. Additionally, conflicting evidence exists on the types of training (high-intensity vs. traditional program). There is a large multi-center study in Australia currently in recruitment to assess high-intensity aerobic training compared to traditional PR (NCT03800914 http://clinicaltrials.gov/show/NCT03800914, accessed on 12 January 2024) [26].

The main barrier to the wide adoption of PR in patients with IPF is undeniably its limited availability; in the United States, easy access to PR is not guaranteed. There are generally not enough programs in rural areas with low population density, and in busy metropolitan areas there are also shortages of spots in PR programs [27]. The COVID-19 pandemic exacerbated these problems by increasing the number of patients needing PR while reducing class sizes to allow for social distancing and forcing shut downs of programs because of insufficient funding. Telehealth-based options were developed to address these deficiencies; the feasibility of this way of providing rehab services has been proven in multiple studies with COPD and post-inflammatory ILD. There have yet to be any data specifically for individuals with IPF [28,29].

## 4. Telehealth and Remote Monitoring

Remote monitoring has attracted interest for a variety of chronic conditions, including IPF. Telehealth programs have been shown to be feasible and attractive to both patients and providers; however, there are no high-quality data indicating whether they can replace routine in-office monitoring, increase medication adherence, or help detect acute exacerbations early [30,31,32]. Moreover, there are concerns around cost, availability, and usability among a population of mostly elderly patients who have traditionally had difficulties adjusting to newer technologies. Further studies are required to further define the type, setting, and population subgroup for which these technologies can provide the most benefit.

## 5. Sleep-Related Hypoxia

Sleep-related breathing disorders are found frequently in patients with IPF; obstructive sleep apnea (OSA) is reported at rates of up to 88% of IPF patients, central sleep apnea (CSA) prevalence rates are reported at 19–22%, and nocturnal hypoxemia (NH), also called sleep-sustained hypoxia (total sleep time with SpO2 under 88% > 5 min and AHI < 15/h), which is not related to obstructive central apnea, is reported in up to 12% of IPF patients [33,34]. The recognition and treatment of these disorders are important, as prolonged nocturnal hypoxia (regardless of etiology, i.e., obstructive vs. nonobstructive pathology) predicts higher mortality [35,36,37]. Treatment with positive pressure therapy and oxygen when indicated may improve mortality [35,37,38]. Many authors advocate for routine polysomnogram screening in all IPF patients given this evidence; furthermore, adherence to therapy with sleep-disordered breathing and nocturnal hypoxia is acceptable in patients with IPF (with varying rates of non-adherence reported in different articles) [34,39,40]. Serial polysomnography is advised over time, as several studies reveal that upon follow-up polysomnography, therapies may need to be added to prior nocturnal prescriptions (i.e., addition of oxygen to PAP or addition of PAP to oxygen therapy). However, the limited availability of sleep specialists makes the implementation of this recommendation challenging.

## 6. Gastroesophageal Reflux Control

Prior studies have demonstrated that up to 87–94% of patients with idiopathic pulmonary fibrosis (IPF) exhibit abnormal esophageal acid exposure, measured through ambulatory esophageal pH monitoring [41,42]. In 2015, guidelines recommended that all patients with IPF should receive pharmacologic GERD treatment, as at that time there was evidence supporting the improvement of respiratory outcomes [43]. However, more recent guidelines have rescinded this recommendation, as evidence for GERD treatment’s effect on pulmonary function testing has not been robust and there have been some concerns about an increase in the risk of respiratory infections [44,45,46]. The association between GERD and IPF, including the role of GERD as a possible causative agent in IPF progression, is a complex and controversial topic. There appears to be an association between the two entities, but a causal relationship between the two has yet to be overtly established [47,48,49].

The initial recommendation for treatment of all patients with IPF with anti-reflux medications has been replaced with a more targeted approach towards patients with IPF and confirmed GERD, as further analyses failed to verify a benefit from global anti-reflux therapies [44,50]. An arsenal of approaches can be attempted, usually in combination, including medications, lifestyle and dietary modifications, and, in select cases, surgical intervention. Lifestyle and dietary modifications can be valuable adjuncts in managing GERD symptoms. Dietary recommendations emphasize educating patients on avoiding foods and beverages known to provoke reflux and encouraging cessation of eating at least 2–3 h before bedtime [51]. Sleep positioning is also essential; elevating the head of the bed and sleeping in a left lateral decubitus position can help prevent reflux by leveraging gravity to keep stomach contents from traveling up the esophagus. Additionally, smoking cessation and weight loss are recommended to further reduce reflux [52]. 

Surgical treatment, such as laparoscopic anti-reflux surgery, has not yet demonstrated a definitive impact on mortality or lung function in IPF patients with GERD [44]. Current American Thoracic Society (ATS) guidelines do not recommend anti-reflux surgery for the improvement of pulmonary outcomes in IPF, which were also summarized by the panel’s meta-analysis [44,50]. However, surgery may be considered in cases of refractory GERD symptoms despite optimal medical management, as the WRAP-IPF trial found this approach to be safe and well-tolerated in this patient population [53].

## 7. Nutrition

Mounting evidence supports the interplay of nutrition with regard to age-related chronic diseases; observations of the interplay of aging and the development of pulmonary fibrosis have also spurned research into the role of diet and nutrition in the disease course of pulmonary fibrosis. Furthermore, patients with IPF commonly have comorbidities that often have nutritional counseling as part of their treatment strategies, such as cardiovascular disease, obesity, diabetes mellitus, and dyslipidemia [46,54,55]. In reference to IPF, the composition of the diet may be important in relation to disease development and progression. Two thought-provoking case-control studies in the Japanese population of IPF patients described diets higher in consumption of meat and saturated fats and lower in consumption of fruits and vegetables [56,57].

The effect of diet composition on lung inflammation has been further evaluated in mouse models; mice exposed to high-fat diets had higher amounts of age-related lung inflammation, mitochondrial deterioration, and decreased lung stem cell populations when compared to mice on calorie-restrictive or low-fat diets [58,59]. Furthermore, measured inflammation in the mice was able to be reduced once the animals’ diets were switched to a low-fat diet [59]. There is not yet enough information on the use of antioxidant supplements in IPF; however, one study noted that combination therapy of vitamins D, C, and E showed improvement in FEV1 and TLC and reduction in systemic inflammation [60].

With respect to measurements of obesity, it has been shown that patients with a lower BMI (body mass index) have increased 3–5-year mortality [61,62,63]. However, data have shown that higher BMI is an independent risk factor for the development of IPF, although it may contribute to decreased mortality, the “obesity paradox” [62]. This paradox has been seen in other respiratory and non-respiratory diseases, such as COPD and heart failure. One of the current theories postulates that the protective factor associated with higher BMI is due to increased lean muscle density, which is not accurately assessed when using the BMI scale and thus is not an adequate prognostic measurement. Interestingly, when comparing the impact of cardio respiratory fitness (CRF) and obesity on mortality in heart failure and COPD, patients with good CRF had decreased mortality regardless of BMI status [64]. Future studies are needed to evaluate the role of body composition and outcomes in IPF as well as diet/nutrition as they relate to progression of disease. As of now, the interplay of nutrition and diet in patients with IPF is a recognized gap in the IPF research knowledge base.

## 8. Lung Cancer Screening

It is well-established that patients with idiopathic pulmonary fibrosis have a very significant risk of developing lung cancer, which is higher than that seen among patients with chronic obstructive pulmonary disease [65]. Moreover, patients with fibrotic ILD have a higher risk of complications, including radiation pneumonitis and chemotherapy-induced pneumonitis, and they are at a significant risk of developing IPF exacerbation after surgical resection [66,67].

Lung cancer screening has been proposed in patients with IPF to promote early detection and treatment [68]. Indeed, among suitable patients with IPF and early-stage lung cancer, surgical resection has been associated with acceptable long-term outcomes [69]. However, the value of early detection may be lower among patients who have more advanced disease and would not be a surgical candidate. In our practice, we perform yearly chest computed tomography among our patients with IPF, and we follow the Fleischner criteria to determine the need for follow-up vs. positron emission tomography or tissue sampling [70].

## 9. Symptom Control and Palliative Care

Palliative experts have described the progression of fibrotic lung diseases as comparable to the progression of malignancy due to the severity of the disease and the effect on QoL. Several studies have demonstrated that the inclusion of palliative care reduces symptom burden and improves quality of life [71]. Patients with IPF suffer not only from direct respiratory symptoms, profound breathlessness, and cough but also non-respiratory symptoms that can include fatigue, anorexia, anxiety, and depression [55]. The non-respiratory symptoms can significantly impact morbidity, with a reported difference in survival of 66 months (without non-respiratory comorbidities) compared to 35 months (with non-respiratory comorbidities) [46]. Unfortunately, non-respiratory symptoms are more difficult to control than direct respiratory symptoms.

To improve QoL, multiple studies, as well as a 2017 meta-analysis from Brighton et al., have shown that “breathlessness support services” and/or a multidisciplinary palliative team, including palliative physicians, respiratory therapists, and nurses, when working with severe fibrotic lung disease result in improvement of breathlessness as well as decreased overall distress related to the disease itself and associated symptoms [72,73,74]. Unfortunately, the impact of palliative multidisciplinary groups has not been as effective for all QoL metrics, including, most notably, anxiety and depression. Although the aforementioned studies saw some improvement in these outcomes, there have been several additional studies that have shown that involving palliative services did not change the individual’s depression or anxiety and at times showed a trend towards worsening these comorbidities [75,76]. Given the variable progression of IPF, optimal timing when introducing palliative services is difficult to determine.

There is some concern that patients recognize the addition of palliative services as discontinuation of life-sustaining care or anti-fibrotic therapies, which is strongly discouraged by palliative groups [71]. This phenomenon has been seen in other chronic terminal diseases, with one study of individuals with malignancy showing that patients were more receptive to services offered through palliative care when the name was switched to supportive care [77]. Other patient barriers to palliative services are misconceptions about potential limitations of offered therapies (including limitations of transplant availability), cultural/religious practices, and misunderstanding of the goals of palliative care [71]. In addition to patient-related barriers, there are multiple health care system barriers that exist. Major deficiencies exist in the availability of palliative resources, which can be due to patients’ geographic locations, adequate palliative care staffing, and, finally, limited expertise in providing palliative services to patients with non-oncologic-related terminal diseases [55,71,78]. While the benefits in terms of different measures are variable, benefits have been shown, and there is a lack of harm. ATS guidelines currently recommend palliative care consultation as an adjunct to other IPF treatments [44].

## 10. Conclusions

Non-pharmacological therapies are a significant part of the management of IPF. They work together with pharmacotherapy to help maintain patients’ quality of life by improving symptom burden, exercise capacity, and the sensation of well-being (Table 1). Further research is needed to determine the nature and timing of the interventions that can provide the best benefit to the most suitable population.

## Figures and Tables

**Table 1:** Summary of non-pharmacological interventions for the treatment of Idiopathic Pulmonary Fibrosis.

Non-Pharmacological Management of IPF					
Therapy	Quality of Data	Benefits	Quality of Life	Guideline Recommendation Strength	Future Research
Supplemental Oxygen	Low	Increased exercise capacity and improved levels of dyspnea	Improved	Strong	Investigations into needs for supplemental oxygen
Pulmonary Rehabilitation	Low	Increased exercise capacity and improved levels of dyspnea	Improved	Weak	Form of Pulmonary Rehabilitation Use of High-Flow oxygen devices during Pulmonary Rehabilitation Role of alternative models of Pulmonary Rehabilitation (Home or telehealth programs)
Telehealth/Remote Monitoring	Emerging	Potential role of improving medication compliance and early detection of exacerbation; Increased perceived support from patients	-	-	Identification of characteristics of patient’s who would benefit from this intervention
Treatment of Sleep Related Hypoxia	High	Decreased nocturnal hypoxemia	-	-	Role of repeated polysomnography
GERD Management	Low	No improvement in pulmonary function If GERD is diagnosed improved GERD symptoms	No improvement in patients without symptoms of GERD	Conditional	Trials powered to evaluate pulmonary functionTrials investigating treatment in IPF population with and without GERD symptoms
Nutrition	Emerging	Negative correlation of BMI and mortality	-	-	Role of nutrition and registered dietician on disease progression
Lung Cancer Screening	High	Early detection of Lung cancer	-	Strong	-
Palliative Care	Low	Improvement in treatment related side effects and comorbid conditions Improved dyspnea Conflicting evidence of benefits of anxiety and depression	Improved	Recommended	Establishment of accepted triggers to initiate Palliative Care Consultation

GERD: Gastric Esophageal Reflux Disease.
